# Efficacy and safety of macitentan for pulmonary hypertension: A meta‐analysis

**DOI:** 10.1111/crj.13621

**Published:** 2023-07-10

**Authors:** Dan Du, Ya‐Dong Yuan

**Affiliations:** ^1^ Department of Respiratory and Critical Care Medicine The Affiliated Hospital of Guizhou Medical University Guiyang Guizhou China; ^2^ Department of Respiratory and Critical Care Medicine The Second Hospital of Hebei Medical University Shijiazhuang Hebei China

**Keywords:** efficacy, macitentan, meta‐analysis, pulmonary hypertension, safety

## Abstract

**Introduction:**

Our purpose of this study is to evaluate the effect and safety of macitentan in the treatment of pulmonary hypertension (PH).

**Methods:**

We retrieved the safety and efficacy of macitentan treatment for PH using PubMed, the Cochrane Library, EMBASE databases and clinicaltrials.gov. The Cochrane Risk of Bias Tool was used for literature screening and quality assessment. Data analysis was conducted using RevMan 5.4.1 and Stata/SE 15.1 software. Results are presented as standardization mean differences (SMDs) and odds ratio (OR).

**Results:**

Meta‐analysis of seven randomized controlled trial (RCT) studies and four non‐RCT studies with 2769 patients was included, involving 723 in the macitentan group and 599 in the placebo group. The results of the study showed that macitentan had effectively decreased pulmonary vascular resistance (PVR) (SMD = −0.53, 95% CI: −0.77–−0.29, *p* < 0.05), cardiac index (CI) (SMD = 0.60, 95% CI: 0.37–0.83, *p* < 0.05) and N‐terminal pro‐brain natriuretic peptide (NT‐proBNP) (SMD = −0.22, 95% CI: −0.40–−0.03, *p* < 0.05). Furthermore, macitentan also significantly reduced PVR (SMD = −0.58, 95% CI: −0.80–−0.35, *p* < 0.05), 6‐min walk distance (6WMD) (SMD = 0.33, 95% CI: 0.15–0.50, *p* < 0.05), CI (SMD = 0.48, 95% CI: 0.28–0.69, *p* < 0.05), mean pulmonary arterial pressure (mPAP) (SMD = −0.43, 95% CI: −0.64–−0.23, *p* < 0.05) and NT‐proBNP (SMD = −0.55, 95% CI: −1.07–−0.03, *p* < 0.05) between baseline and follow‐up. The adverse reactions to macitentan were mild, with headache, anaemia and bronchitis. Other efficacy and safety outcomes did not reach statistical differences.

**Conclusion:**

Macitentan therapy for PH is effective and safe. The effectiveness on PVR, mPAP, mean right atrial pressure (mRAP), mortality and other indicators still needs to be further confirmed.

## Graphical abstract

Macitentan therapy for pulmonary hypertension is effective and safe. The effectiveness on PVR, mPAP, mRAP, mortality and other indicators still needs to be further confirmed.

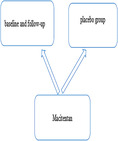

## 1 INTRODUCTION

Pulmonary hypertension (PH) is an uncommon, devastating and progressive disorder, leading to increases in pulmonary vascular remodelling. Right heart failure as an end‐stage of PH threatens death.^1^ Of all vasodilators, endothelin receptor antagonists, which are strong vasodilators, are capable of stopping the process of cell division, which could remodel pulmonary arterial structural alteration.^2^

For more than 30 years, endothelin was first reported as a vasoconstrictor.^3^ The endothelin receptor antagonist (ERA) receptor subtypes include dual antagonists that block ETA and ETB receptors. The dual ERA bosentan was approved as the first oral therapy for PH.^4^, ^5^ However, there was an increased incidence of dose‐dependent liver transaminase and peripheral edema. Macitentan is the result of this optimization programme, macitentan, an endothelin receptor antagonist of the third generation, has been approved for long‐term treatment of PH.^6^ Animal experiments showed that treatment with macitentan improved haemodynamic parameters in a monocrotaline model of PH.^7^

In an increasing number of clinical studies, marcitentan has shown remarkable efficacy in different types of PH. The SERAPHIN trial was a landmark trial with a large number of enrolled patients and a long observation period, which significantly reduced morbidity and mortality in patients with PH. It has also been shown to improve haemodynamic pulmonary vascular resistance (PVR) and performance in Eisenmenger syndrome‐associated pulmonary arterial hypertensions (PAHs), as well as 6‐min walk distance (6WMD) and PVR in inoperable chronic thromboembolic PH and left ventricular insufficiency. Meanwhile, the incidence of adverse events such as hepatotoxicity and edema was lower than that of other ERAs.

Nowadays, there is a lack of systematic reviews on macitentan's efficacy and safety in the treatment of PH patients. Thus, a systematic review to determine the efficacy of macitentan in the treatment of PH was necessary. We are searching for relevant literature from the PubMed, EMBASE, Cochrane Library databases and clinicaltrials.gov.

## 2 THE METHODS INVOLVED

### 2.1 Data collection and management

#### 2.1.1 Inclusion criteria

1. Type of literature: full‐text published literature to study the therapeutic effect of macitentan on patients with PH through randomized controlled and observational studies;
2. The establishment time of the selected literature: June 2022;
3. Research object: Adult patients with PH or any recognized diagnostic criteria, no macitentan was taken before the study, and regular medication and timely follow‐up were conducted after the study;
4. Intervention: the experimental group was treated with macitentan under the same conditions as the control group, or clinical data obtained after macitentan treatment for PH were compared with baseline follow‐up;
5. Outcome measures: case fatality rate during treatment and follow‐up, 6WMD, PVR, cardiac index (CI), mean pulmonary arterial pressure (mPAP), mean right atrial pressure (mRAP), mixed venous oxygen saturation (SvO2) and common adverse reactions.

#### 2.1.2 Exclusion criteria

1. The subjects of this study were not PH patients; they took macitentan before participating in the study; they used drugs irregularly after the start of the study and were lost to follow‐up;
2. The literature includes reviews, case reports, pharmacological studies and animal studies;
3. Repetition of the published articles;
4. The literature provides partial missing data or data from which outcome indicators cannot be extracted;
5. The full text of the literature cannot be consulted.

#### 2.1.3 Qualifications for exclusion

1. In this study, the patients were not PH patients who took macitentan before participating in the study. Instead, they used the drug irregularly after the study began and lost contact with the study's investigators;
2. Studies in non‐human experimentation, case reports and pharmacological studies;
3. Article duplication;
4. Missing data or data from which outcome indicators cannot be derived;
5. Literature cannot be consulted in full.

#### 2.1.4 Data retrieval strategy

**FIGURE 1:**
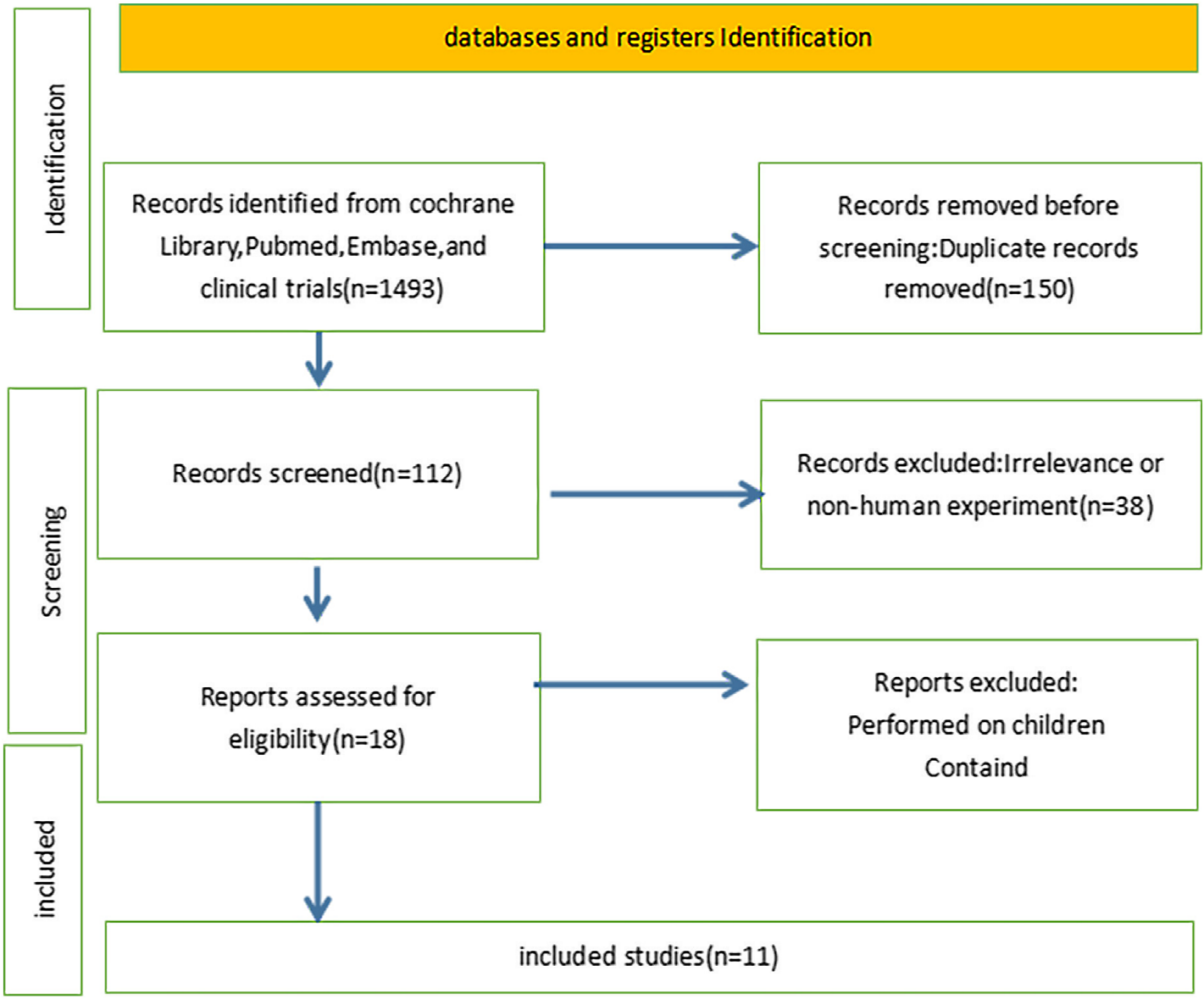
Literature retrieve flow diagram.

The required databases, PubMed, EMBASE and clinical trials, were reviewed and extracted from the literature from the date of establishment to June 2022. Each study was as follows: the first author, year of publication, study population, intervention, race, age, sample size, research technique, main outcome, follow‐up time and adverse reactions. The terms included free words and medical subject headings (MeSH) subject words. The English literature can be retrieved with the key words (‘pulmonary arterial hypertension’ OR ‘pulmonary hypertension’ OR ‘pulmonary artery hypertension’ OR ‘pulmonary vascular disease’ OR ‘pulmonary heart disease’ OR ‘pulmonary cardiac disease’ OR PAH) AND (macitentan OR N‐(5‐(4‐bromophenyl)‐6‐(2‐(5‐bromopyrimidin‐2‐yloxy)ethoxy)pyrimidin‐4‐yl)‐N′‐propylaminosulfonamide OR ACT 064992 OR ACT064992 OR ACT‐064992 OR Actelion‐1 OR opsumit) AND (clinical trials). In sum, 11 English literature were located. Figure 1 shows the detailed literature screening process.

#### 2.1.5 Literature screening and data extraction

After the literature was screened from the database, EndNote X9 was used to manage the screened literature, and duplicate studies were excluded using the software. Two researchers then screened the studies separately. In the initial screening, irrelevant articles were excluded according to the standard by reading the titles and abstracts of each article. During the second screening, the full text of the articles was reviewed, and articles for which the outcome data could not be extracted were excluded. After the second screening, the relevant data from the included literature were extracted. Data extracted included the first author, year of publication, study population, race, age, sample size, research method, main outcome, follow‐up time and adverse reactions.

#### 2.1.6 Literature quality assessment

The Cochrane Bias Risk Assessment Table and Newcastle–Ottawa Scale were used by two researchers as bias risk assessment tools in this study to evaluate the quality of the literature. When opinions differ, the decision is reached through discussion or based on the third investigator's intervention. The evaluation indicates that the study by Nobuhiro Tahara, Pankaj Jariwala, Pavel Jansa, Satomi Sakural and Tomas Pulido exhibits high‐risk random, allocation and binding biases. Randomized controlled trial (RCT) studies have low‐risk random bias, allocation bias, and binding bias. In addition, in all studies, there is an unclear risk of selection bias due to insufficient descriptions of details.

### 2.2 Analysing the quality of literature

Based on the Cochrane Collaboration methodology, two researchers independently assessed the quality of the literature for bias in meta‐analysis, including selection bias and biases in the performance, detection, reporting and analysis of data. Every risk of bias has three levels of uncertainty: low, medium and high.

### 2.3 Statistical analysis

All analysis data were performed with RevMan version 5.4.1 and Stata/SE 15.1. The study was assessed using a random‐effects model, and the included study was assessed as statistically heterogeneous by I^2^ with a value exceeding 50%. Otherwise, the fixed‐effects model is available. As a result, heterogeneity was considered acceptable. Sensitivity analyses were conducted to assess whether the combined results would be significantly affected by a single study, and funnel plots were used to assess publication bias.^8^, ^9^

## 3 RESULTS

### 3.1 Basic information of included literature

The flow chart of the literature search and selection is shown in Figure 1. We retrieved 2769 citations in total. After screening for titles and abstracts, 150 records were excluded because of irrelevance or noncontrolled design. In total, 112 articles remained for further evaluation. Among them, 38 were excluded. Ultimately, we identified seven RCTs, one prospective study, one retrospective study and two cohort studies.

### 3.2 Search results

Eleven studies completely fulfilled the criteria in the literature, including the studies by Hossein‐Ardeschir Ghofrani,^10^ Jean‐Luc Vachiéry,^11^ Michael A. Gatzoulis,^12^ Nazzareno Galie^13^ and Prof. Olivier Sitbon,^14^ and non‐RCTs are Nobuhiro Tahara,^15^ Pavel Jansa,^16^ Nobuhiro Tahara,^17^ Satomi Sakurai,^18^ Pankaj Jariwala^19^ and Tomás Pulido.^20^

Seven RCT studies focus on a multicentre, double‐blind, randomized, parallel group, placebo‐controlled, Hossein‐Ardeschir Ghofrani study. Following the open‐label treatment period, all eligible patients received macitentan 10 mg for 24 weeks. Jean‐Luc Vachiéry's study was on left ventricular dysfunction. The macitentan treatment period, therefore, 12 weeks for a follow‐up, was the main end of fluid retention or New York Heart Association (NYHA) functional. In Michael A. Gatzoulis's study of Eisenmenger syndrome, clinical assessments and laboratory tests were done at macitentan 10 mg for the double‐blind and open‐label treatment periods, respectively. Nazzareno Galie's study of PAH was done at week 12. A 28‐day screening period was followed by a 1:1:1 randomization in which patients received once daily either macitentan 3 mg, macitentan 10 mg or a placebo. Patients received double‐blind treatment until they experienced a primary endpoint event, including a series of haemodynamics, CI, mRAP, mPAP, PVR, SVO2 and N‐terminal pro‐brain natriuretic peptide (NT‐proBNP). In the study of Prof. Olivier Sitbon, in this phase 4 trial, PH was treated with double‐blind, placebo‐controlled, multicentre treatments over 12 weeks. Satomi Sakurai's study of PH was a prospective, multicentre, open‐label, single‐arm, phase 3 study with PVR, 6WMD, CI, CO, mRAP, SVO2 and NT‐proBNP. Five RCT studies included morbidity and mortality as main ending (Table 1).

**TABLE 1:** Included literature information.

Author	Year of publication	Research type	Age (mean/median)	Sample size	Grouping	Combination or monotherapy	Study population	Follow‐up time	Main ending
Hossein‐Ardeschir Ghofrani^10^	2017	RCT	57.5	80	macitentan (10 mg)	Monotherapy	CTEPH	24 weeks	PVR
Jean‐Luc Vachiéry^11^	2018	RCT	71	63	macitentan (10 mg)	Monotherapy	LHD‐PH	12 weeks	mRAP
Michael A. Gatzoulis^12^	2019	RCT	32	226	macitentan (10 mg)	Monotherapy	LHD‐PH	16 weeks	6WMD
Nazzareno Galie^13^	2017	RCT	47	742	macitentan (10 mg)	Monotherapy	PAH	24 weeks	CI, mRAP, mPAP, PVR, SVO2, NT‐proBNP
Prof. Olivier Sitbon^14^	2019	RCT	58.5	85	macitentan (10 mg)	Monotherapy	PPHTN	12 weeks	PVR
Nobuhiro Tahara^15^	2020	COH	49	30	macitentan (10 mg)	Monotherapy	PAH	24 weeks	6WMD, NT‐proBNP
Pavel Jansa^16^	2017	RCT	46	742	macitentan (10 mg) + PDE‐5i	Combination	IPAH, HPAH	24 weeks	Morbidity mortality
Nobuhiro Tahara^17^	2016	COH	49	30	macitentan (10 mg)	Monotherapy	PAH	24 weeks	6WMD, NT‐proBNP, PVR
Satomi Sakurai^18^	2021	PRO	69	9	macitentan (10 mg)	Monotherapy	CTEPH	16 weeks	PVR, 6WMD, CI, CO, mRAP, SVO2, NT‐proBNP
Pankaj Jariwala^19^	2022	RET	43.4	20	macitentan (10 mg)	Monotherapy	PH	48 weeks	6WMD, sPAP, NT‐proBNP
Tomas pulido^20^	2013	RCT	45.6	742	macitentan (10 mg)	Monotherapy	PAH	24 weeks	Morbidity mortality

Abbreviations: 6WMD, 6‐min walk distance; CI, cardiac index; CO, cardiac output; COH, cohort study; CTEPH, chronic thromboembolic pulmonary hypertension; HPAH, hereditary PAH; IPAH, idiopathic PAH; LHD, left heart diseases; mRAP, mean right atrial pressure; PAH, pulmonary arterial hypertension; PAP, pulmonary arterial pressure; PH, pulmonary hypertension; PPHTN, portopulmonary hypertension; PRO, prospective study; PVR, pulmonary vascular resistance; RCT, randomized controlled trials; RET, retrospective study.

### 3.3 Bias and quality assessment

**FIGURE 2:**
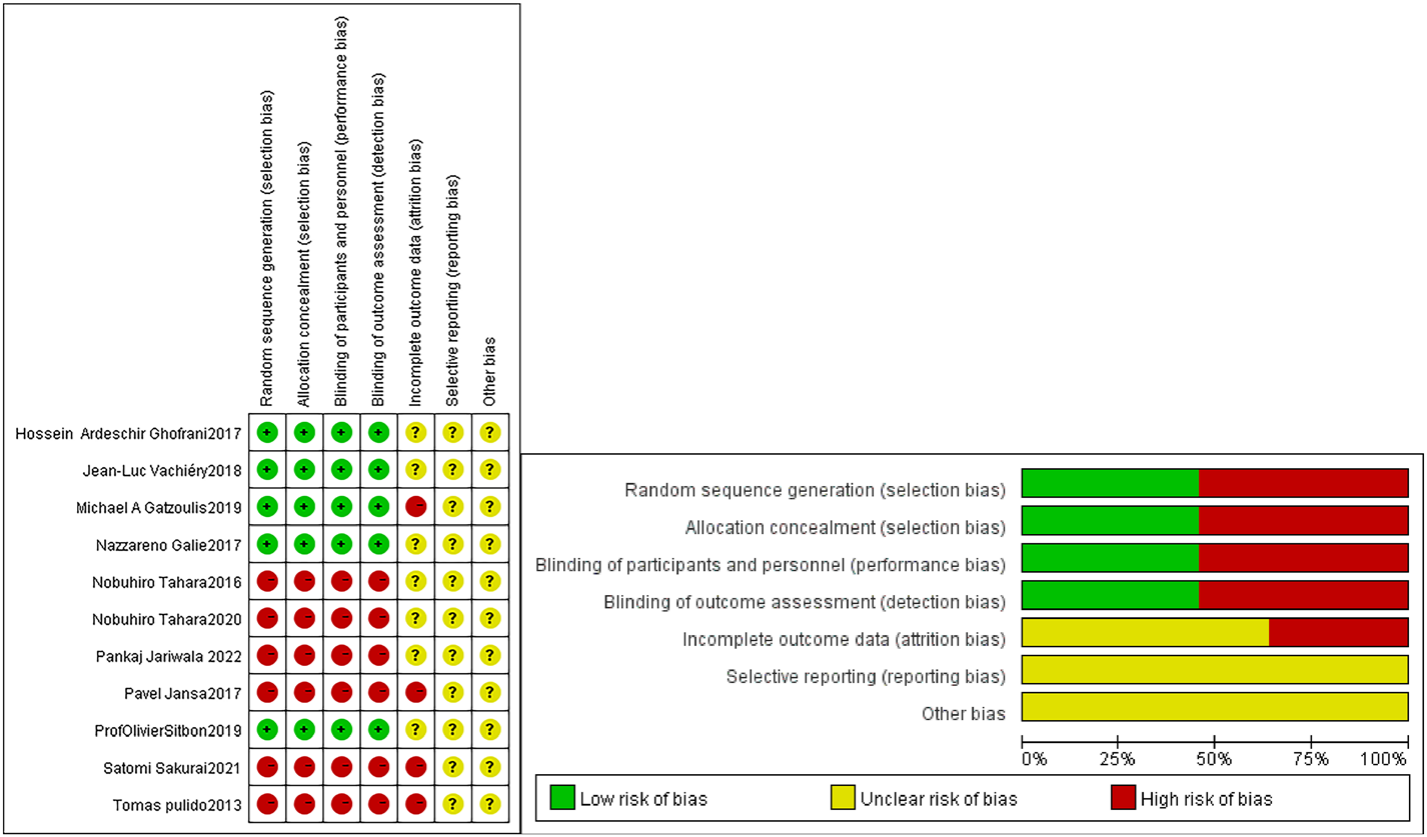
Literature quality assessment.

The results of bias risk evaluation for the enrolled RCTs are shown in Figure 2. Green represents low risk, red represents high risk, and yellow represents unclear risk, and it can be seen that there was no obvious bias in this study.

### 3.4 Effect of macitentan therapy on PH

#### 3.4.1 Change in PVR: macitentan versus placebo

**FIGURE 3:**
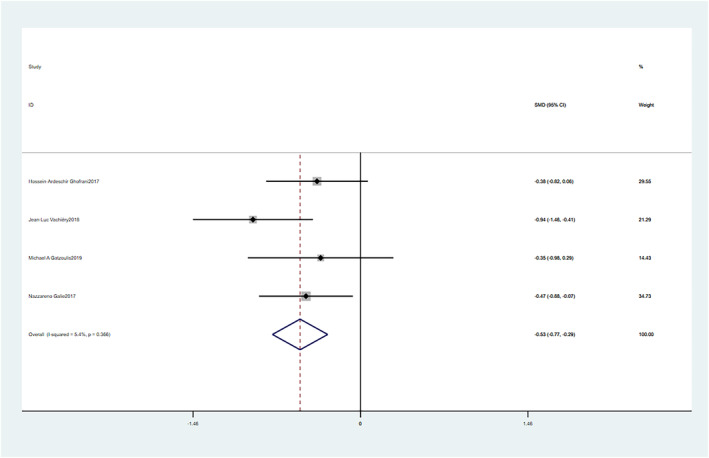
Meta‐analysis of the effects of macitentan versus placebo on pulmonary vascular resistance (PVR).

There was no significant statistical heterogeneity among the results of the studies, and the fixed effects model was used for meta‐analysis. There is no significant statistical heterogeneity among the studies, and the fixed‐effect model was used (SMD = −0.53, 95% CI: −0.77–−0.29, *p* = 0.000). These differences were statistically significant (Figure 3).

#### 3.4.2 Changes in PVR: comparison of baseline and follow‐up of PH

**FIGURE 4:**
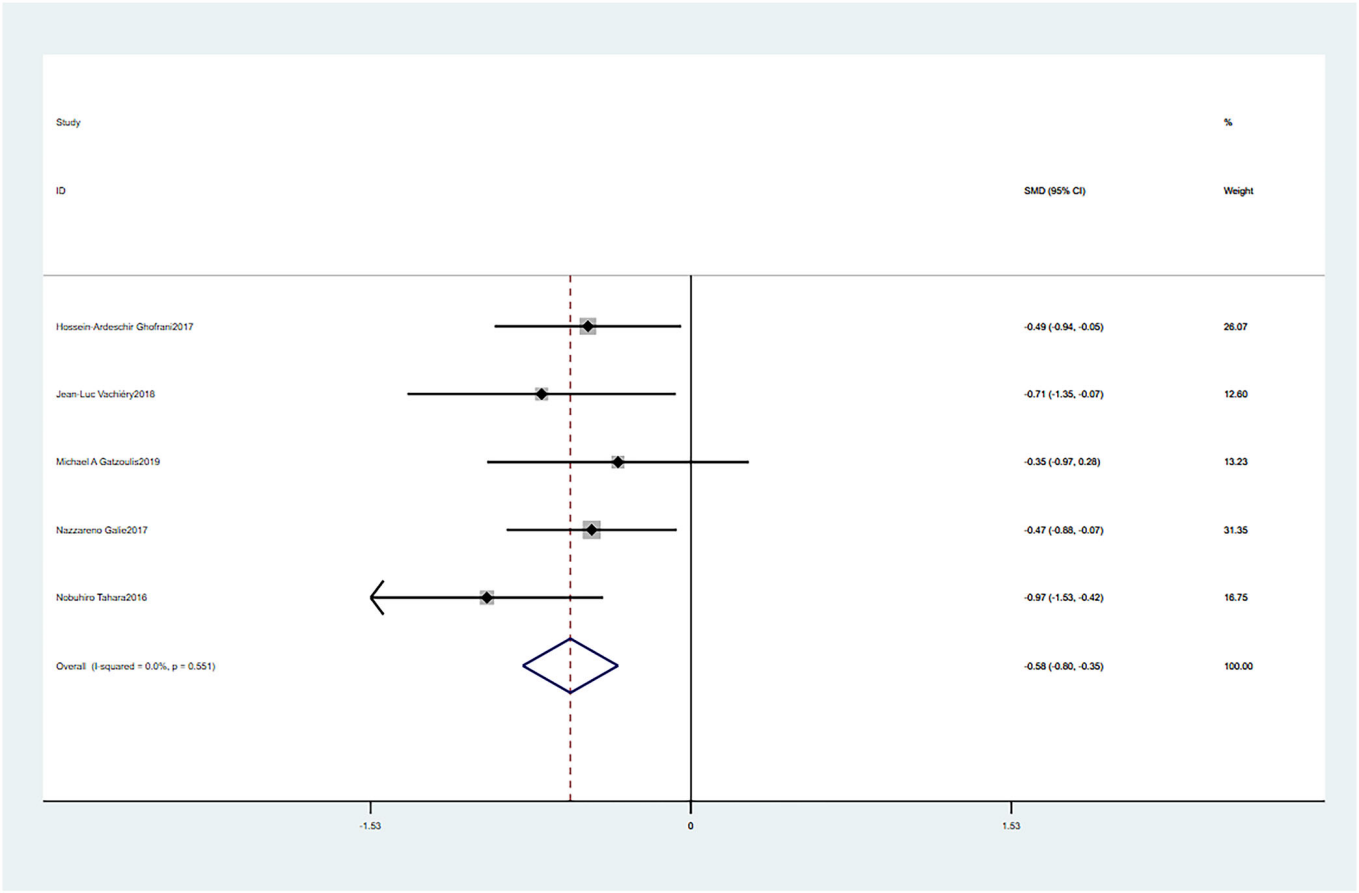
Meta‐analysis of the effects of follow‐up versus baseline on pulmonary vascular resistance (PVR).

Macitentan treatment showed significant improvement in PVR compared to pretreatment baseline measures (SMD = −0.58, 95% CI: −0.80–0.35, *p* = 0.000). The fixed effect model was used (Chi^2^ = 3.04, *p* = 0.551, I^2^ = 0%). These differences were statistically significant (Figure 4).

### 3.5 Changes in 6WMD: macitentan versus placebo

**FIGURE 5:**
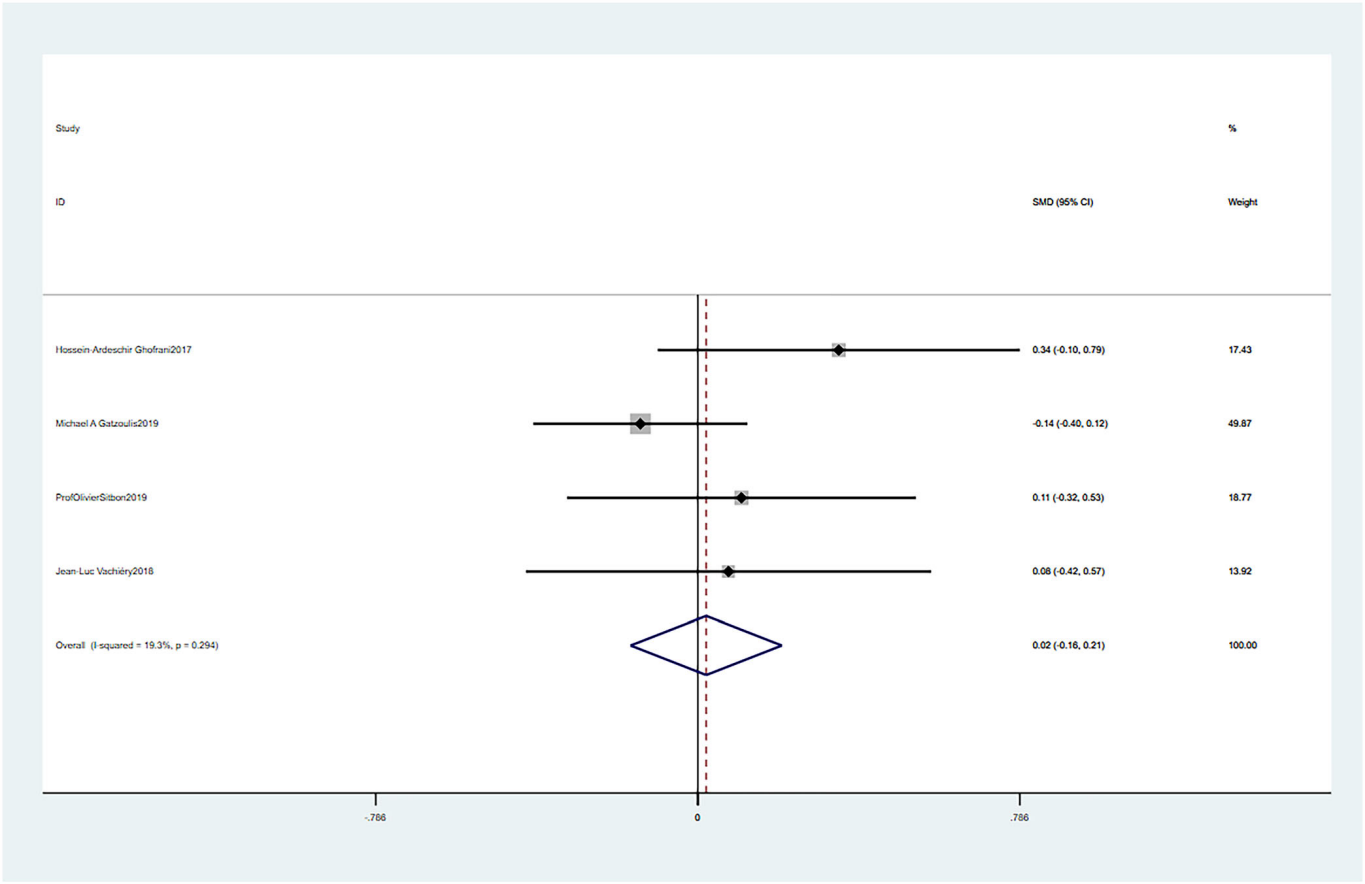
Meta‐analysis of the effects of macitentan versus placebo on 6‐min walk distance (6WMD).

Macitentan treatment demonstrated no significant improvement in 6WMD compared to placebo with a mean difference (SMD = 0.02, 95% CI: −0.16–0.21, *p* = 0.826). The fixed effect model was used (Chi^2^ = 0.15, *p* = 0.294, I^2^ = 19.3%). These differences were statistically significant (Figure 5).

#### 3.5.1 Changes in 6WMD: comparison of baseline and follow‐up of PH

**FIGURE 6:**
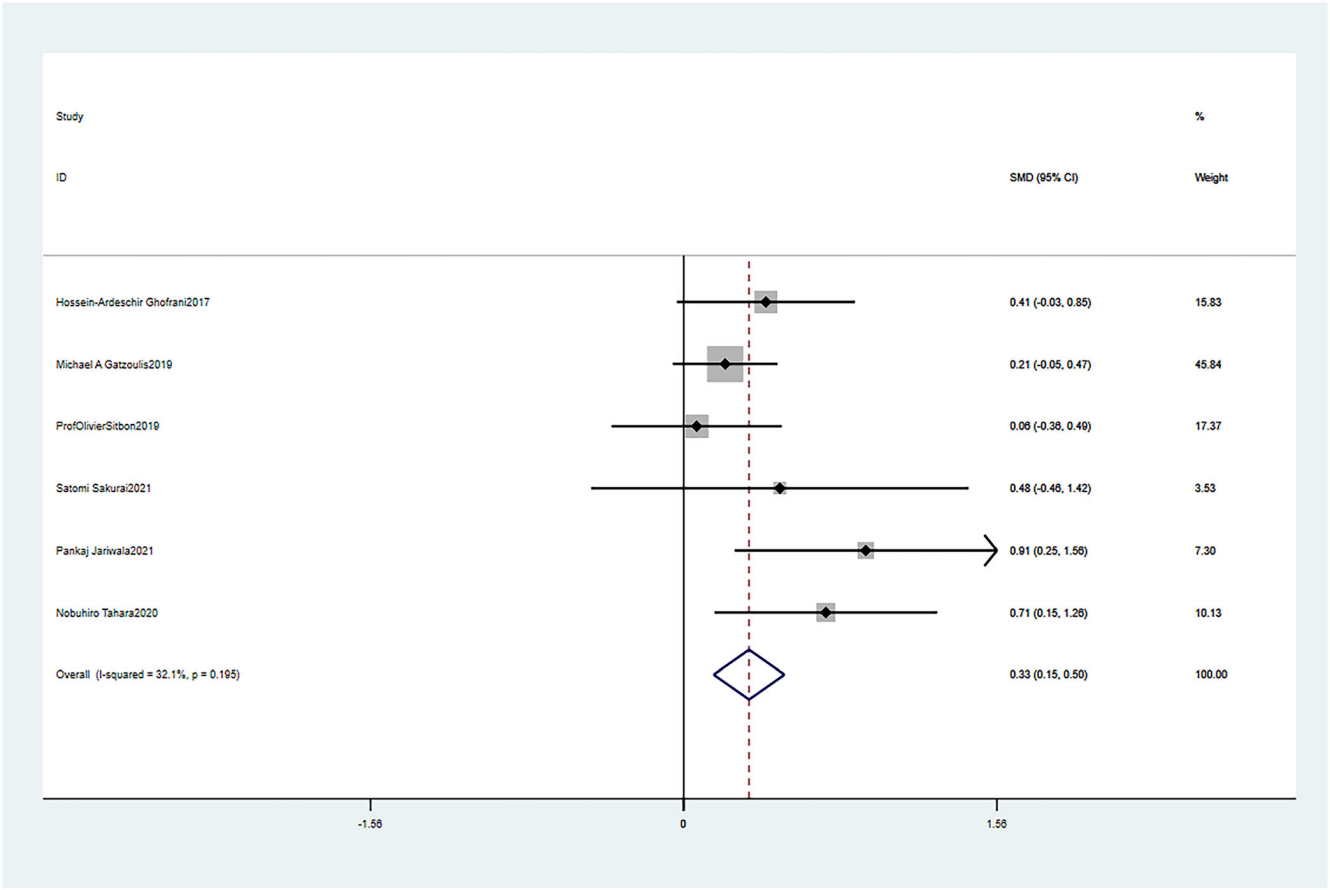
Meta‐analysis of the effects of follow‐up versus baseline on 6‐min walk distance (6WMD).

Patients treated with macitentan significantly improved their 6WMD compared to pretreatment baseline measures (SMD = 0.33, 95% CI: 0.15–0.50, *p* = 0.000). The fixed effect model was used (Chi^2^ = 7.37, *p* = 0.195, I^2^ = 32.1%). These differences were statistically significant (Figure 6).

### 3.6 Changes in mPAP: macitentan versus placebo

**FIGURE 7:**
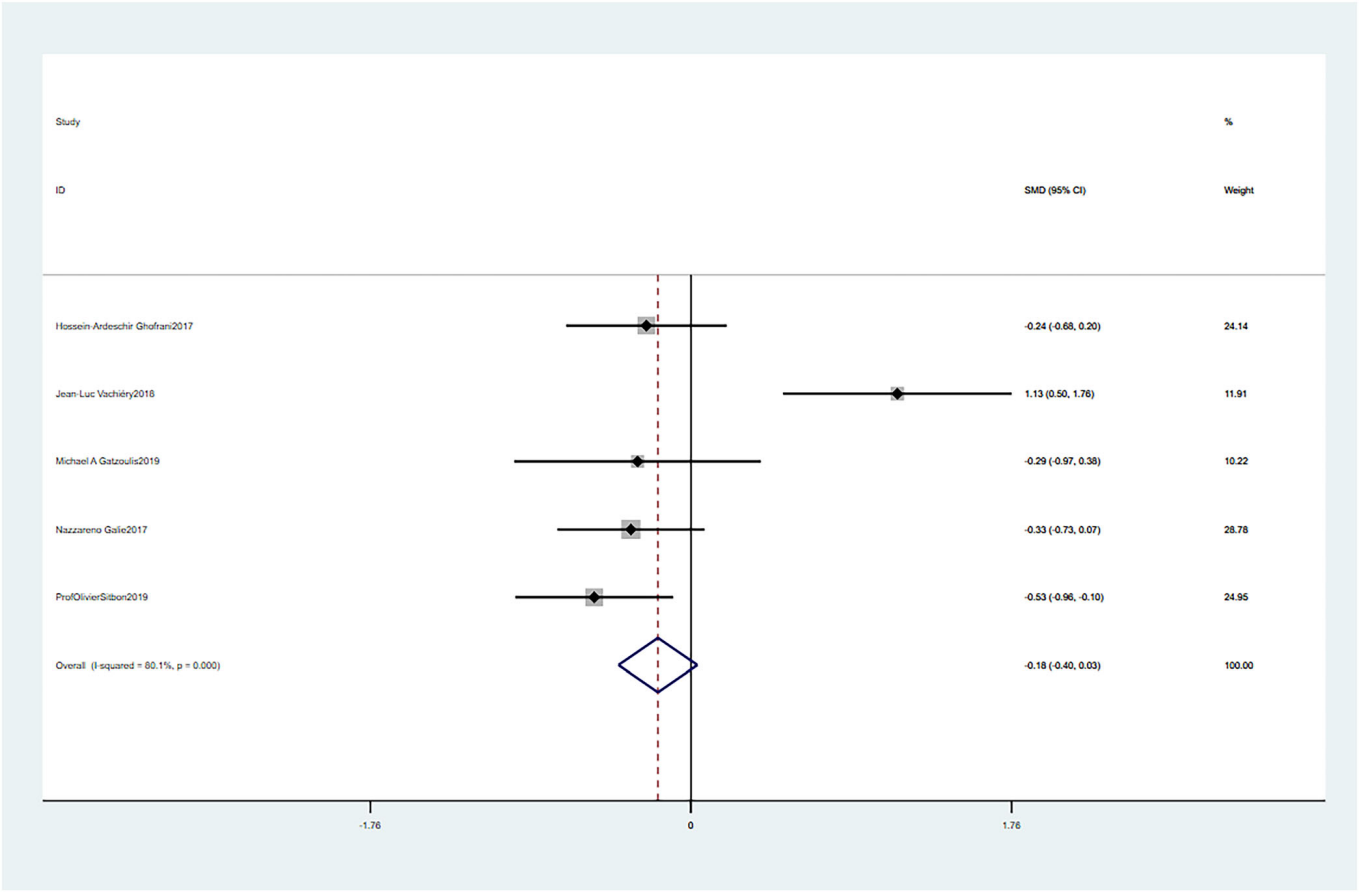
Meta‐analysis of the effects of macitentan versus placebo on mean pulmonary arterial pressure (mPAP).

Macitentan treatment showed no significant improvement in mPAP compared to placebo with a mean difference (SMD = −0.18, 95% CI: −0.40–0.03, *p* = 0.100). There was still significant heterogeneity after article by article exclusion indicating that the heterogeneity was stable (Chi^2^ = 20.05, *p* = 0.000, I^2^ = 80.1%), and the random effect model was used. These differences were not statistically significant (Figure 7).

#### 3.6.1 Changes in mPAP: comparison of baseline and follow‐up of PH

**FIGURE 8:**
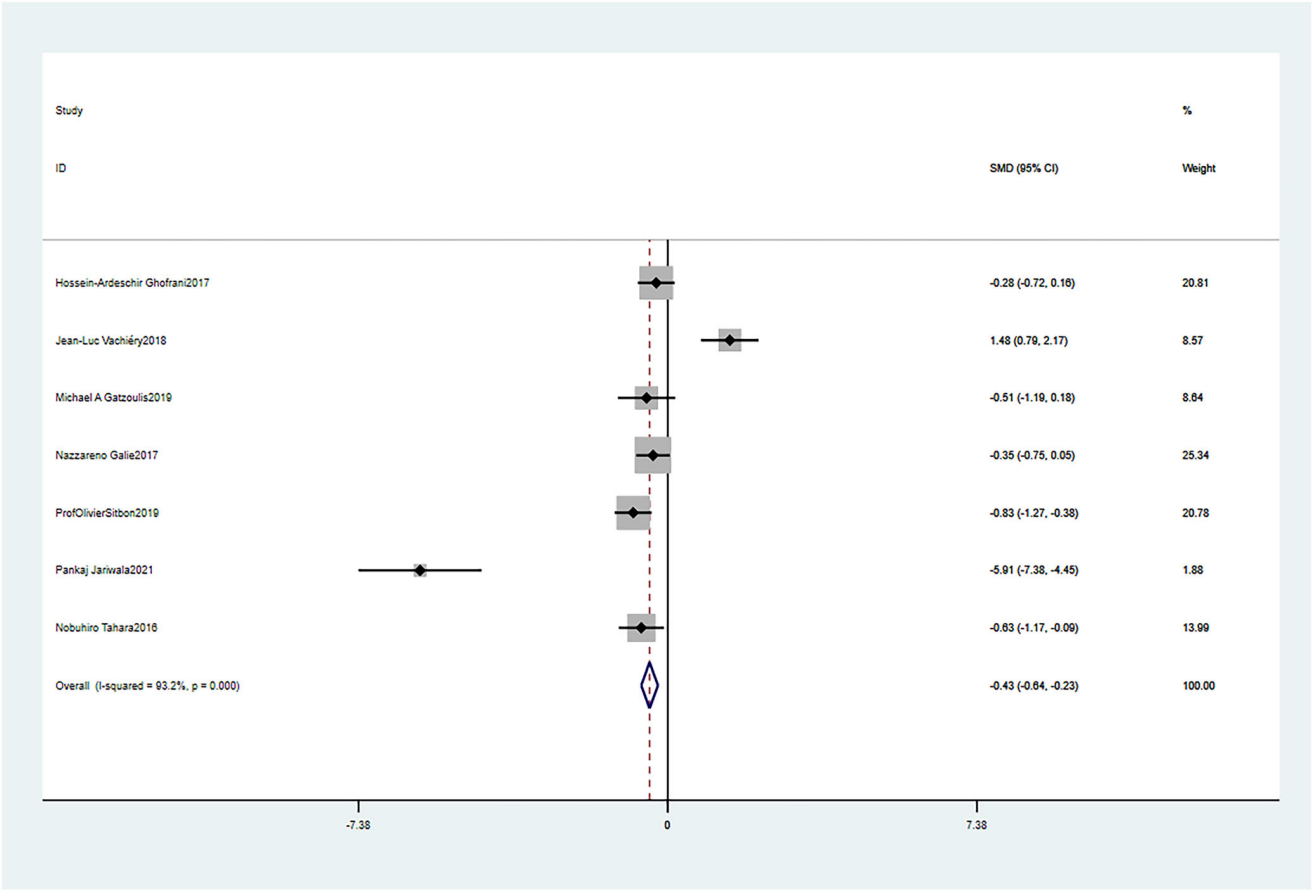
Meta‐analysis of the effects of follow‐up versus baseline on mean pulmonary arterial pressure (mPAP).

There is no significant statistical heterogeneity (*p* < 0.1, I^2^ > 50%) (Chi^2^ = 87.75, *p* = 0.000, I^2^ = 93.2%), and the fixed‐effect model was used (SMD = −0.43, 95% CI: −0.64–−0.23, *p* = 0.000). These differences were not statistically significant (Figure 8).

### 3.7 Changes in mRAP: macitentan versus placebo

Macitentan treatment showed no significant improvement in mPAP compared to placebo with a mean difference (SMD = −0.18, 95% CI: −0.40–0.03, *p* = 0.100). The random effect model was used (Chi^2^ = 20.05, *p* = 0.000, I^2^ = 80.1) There was still significant heterogeneity after article by article exclusion indicating that the heterogeneity was stable. These differences were not statistically significant (Figure S1).

#### 3.7.1 Changes in mRAP: comparison of baseline and follow‐up of PH

Patients treated with macitentan had no significantly improved their mRAP compared to baseline measures (SMD = 0.01, 95% CI: −0.18–0.20, *p* = 0.923). The fixed effect model was used (Chi^2^ = 9.01, *p* = 0.173, I^2^ = 33.4%). These differences were not statistically significant (Figure S2).

### 3.8 Changes in CI: macitentan versus placebo

Macitentan treatment showed a significant improvement in CI compared to placebo with a mean difference (SMD = 0.60, 95% CI: 0.37–0.83, *p* = 0.000). The fixed effect model was used (Chi^2^ = 0.15, *p* = 0.585, I^2^ = 18.6%). These differences were statistically significant (Figure S3).

#### 3.8.1 Changes in CI: comparison of baseline and follow‐up of PH

Patients treated with macitentan significantly improved their CI compared with pretreatment baseline measures (Chi^2^ = 4.73, *p* = 0.450, I^2^ = 0%). No significant statistical heterogeneity was noted among the studies, and the fixed‐effect model was used (SMD = 0.48, 95% CI: 0.28–0.69, *p* = 0.000). These differences were statistically significant (Figure S4).

### 3.9 Changes in NT‐proBNP: macitentan versus placebo

Macitentan treatment showed a significant improvement in NT‐proBNP compared with placebo (Chi^2^ = 4.24, *p* = 0.237, I^2^ = 29.2%). There is no significant statistical heterogeneity among the studies, and the fixed‐effect model was used (SMD = −0.22, 95% CI: −0.40–−0.03, *p* = 0.000). These differences were statistically significant (Figure S5).

#### 3.9.1 Changes in NT‐proBNP: comparison of baseline and follow‐up of PH

Patients treated with macitentan significantly improved their NT‐proBNP compared with pretreatment baseline measures. Significant statistical heterogeneity was noted among the studies, and the random effect model was used (SMD = −0.55, 95% CI: −1.07–−0.03, *p* = 0.000). These differences were statistically significant (Figure S6).

### 3.10 Changes in SVO2: macitentan versus placebo

Macitentan treatment showed a significant improvement in SVO2 compared with placebo (Chi^2^ = 0.15, *p* = 0.294, I^2^ = 19.3%). No significant statistical heterogeneity was noted among the studies, and the fixed‐effect model was used (SMD = 0.11, 95% CI: −0.12–0.34, *p* = 0.338). These differences were not statistically significant (Figure S7).

#### 3.10.1 Changes in SVO2: comparison of baseline and follow‐up of PH

Patients treated with macitentan significantly improved their SVO2 compared with pretreatment baseline measures (Chi^2^ = 0.94, *p* = 0.919, I^2^ = 0%). No significant statistical heterogeneity was noted among the studies, and the fixed‐effect model was used (SMD = 0.12, 95% CI: −0.09–0.34, *p* = 0.250). These differences were not statistically significant (Figure S8).

### 3.11 Mortality in PH

Macitentan treatment was associated with no significant reduction in mortality compared to placebo (OR = 1.20, 95% CI: 0.80–1.79, *p* = 0.37). The fixed effect model was used (Chi^2^ = 1.80, *p* = 0.77, I^2^ = 0%) (Figure S9).

### 3.12 Common adverse reactions

Common adverse reactions refer to peripheral edema, headaches, bronchitis and anaemia. Except for peripheral edema (SMD = 1.33, 95% CI: 0.80–2.18, *p* = 0.27), other symptoms were statistically significant (Figures S10, S11, S12 and S13).

## 4 DISCUSSION

PH is characterized by pulmonary arterial remodelling. At present, if untreated, PH has a median survival of 2 years from the time of diagnosis, and the death outcome is usually right ventricular failure.^21^, ^22^ Drug therapy for PH consists of the nitric oxide, endothelin and prostacyclin pathways. These specific therapies have improved 5‐year survival from 34% in 1991 to more than 60% in 2015.^23^

Endothelin‐1, discovered as endothelial vasoconstrictor peptide as early as 1987,^24^ showed potent and long‐lasting vasoconstrictor effects on arteries that were never observed with another compound at the time. The ETA receptor is dominant in smooth muscle cells and mediates vasoconstriction and cell hypertrophy, whereas the ETB receptor is expressed mainly on the vascular endothelium and mediates vasodilation. The ETA receptor plays a dominant role in vasoconstriction.^25^ Macitentan, which was first approved for PAH in 2013, improves its efficacy and tolerability by altering the structure of bosentan, reducing side effects such as hepatotoxicity and lower limb fluid retention.^26^, ^27^ To our knowledge, the present study is the first, largest and most comprehensive meta‐analysis of the efficacy and safety of macitentan in PH patients, summarizing multiple RCT and non‐RCT studies.

In the included studies, PVR, CI, 6WMD, CI, NT‐proBNP, mPAP, mRAP and SvO2 were used to evaluate the clinical efficacy of macitentan. The results of this study demonstrated that compared with placebo, macitentan significantly reduces PVR (SMD = −0.53, 95% CI: −0.77–−0.29, *p* < 0.05), CI (SMD = 0.60, 95% CI: 0.37–0.83, *p* < 0.05) and NT‐proBNP (SMD = −0.22, 95% CI: −0.40–−0.03, *p* < 0.05). Compared with the baseline outcome, the results also showed that macitentan effectively improves PVR. Compared with placebo and baseline, the results consistently indicate that macitentan effectively increases pulmonary vascular resistance (PVR), the cardiac index (CI) and NT‐proBNP. Whether macitentan effectively alters mPAP, mRAP, SvO2 and 6WMD remains unknown. More studies and clinical data are needed to confirm this notion.

Seven RCTs included articles reporting numerous adverse reactions during macitentan treatment. This study conducted a meta‐analysis of peripheral edema, headache, bronchitis and anaemia. The results showed significant differences between the above adverse reactions and placebo, but the results did not indicate that macitentan treatment increases the incidence of peripheral edema. Headaches, bronchitis and anaemia are the most common adverse reactions, and most patients tolerate them.

Our study has several limitations. Firstly, there are few included literature from RCTs, which may impact the reliability of the results. The results indicate that macitentan treatment affects PVR, CI and NT‐proBNP. In the future, even more subjects will enroll in RCT studies to evaluate efficacy and safety. Secondly, the studies included in the meta‐analysis were heterogeneous compared with macitentan and placebo. A sensitivity analysis was available. No single study had a significant impact on combining results. So, the results are relatively stable. We could not conduct further subgroup analysis because of the limited data. Finally, the WHO functional class is scarcity.

In conclusion, this meta‐analysis showed that macitentan therapy efficiently improved symptoms and haemodynamics in patients with PH in terms of CI, PVR and NT‐proBNP. However, macitentan did not provide benefits, including mPAP, mRAP and SvO2. It remains uncertain whether macitentan has an impact on 6WMD. In future studies, the effect of macitentan on other cardiopulmonary function measures will need to be confirmed. In addition, macitentan therapy was safe and well tolerated. Although the long‐term efficacy and safety of the medication must be more fully established, macitentan confers therapeutic benefits in patients with PAH. The effectiveness on PVR, mPAP, mRAP, mortality and other indicators still needs to be further confirmed.

## AUTHOR CONTRIBUTIONS

Ya‐Dong Yuan. designed the study. Dan Du designed and ran the literature search. Dan Du performed the title and abstract screening. The conflicts were resolved by Ya.Dong.Yuan. The full text review was done by extracting the data and analysing them. Ya.Dong.Yuan. assessed the risk of bias. Dan Du wrote the manuscript. All authors provided critical conceptual input, analysed and interpreted data and critically revised the report. All persons who meet authorship criteria are listed as authors, and all authors certify that they have participated sufficiently in the work to take public responsibility for the content, including participation in the concept, design, analysis, writing, or revision of the manuscript. Furthermore, Each author certifies that this material or similar material has not been and will not be submitted to or published in any other publication before its appearance in The Clinical Respiratory Journal.

## CONFLICT OF INTEREST STATEMENT

All content on this website, including text, pictures, audio, software, programmes and layout design, has been collected from the Internet. Visitors may use the content or services of this site for personal study, research or enjoyment, and for other non‐commercial or non‐profit purposes.

However, it shall comply with the provisions of copyright law and other relevant laws and shall not infringe upon the legitimate rights of the website and relevant right holders. In addition, any use of any content or service of the website for other purposes shall be subject to the written permission of the website and relevant right holders and payment of remuneration. If the original author of the content of this website is not willing to publish the content on this website, please inform the website in time to delete it.

## ETHICAL STATEMENTS

The ethical statements are not applicable in this meta‐analysis.

## Supporting information


**Figure S1.** Meta analysis of the effects of macitentan vs placebo on mRAP.

**Figure S2.** Meta analysis of the effects of Follow‐up vs baseline on mRAP.

**Figure S3.** Meta analysis of the effects of macitentan vs placebo on CI.

**Figure S4.** Meta analysis of the effects of Follow‐up vs baseline on CI.

**Figure S5.** Meta analysis of the effects of macitentan vs placebo on NT‐proBNP.

**Figure S6.** Meta analysis of the effects of Follow‐up vs baseline on NT‐proBNP.

**Figure S7.** Meta analysis of the effects of macitentan vs placebo on SVO2.

**Figure S8.** Meta analysis of the effects of Follow‐up vs baseline on SVO2.

**Figure S9.** Meta analysis of mortality in PH.

**Figure S10.** Meta analysis of incidence of peripheral edema.

**Figure S11.** Meta analysis of incidence of headache.

**Figure S12.** Meta analysis of incidence of bronchitis.

**Figure S13.** Meta analysis of incidence of anaemic events.

Supporting info item

## Data Availability

Research data are not shared.
