# Supplementary figures and images for: Efficacy and safety of macitentan for pulmonary hypertension: A meta‐analysis
Source: Clin Respir J. 2023 Jul 10;17(11):1117–29. doi: 10.1111/crj.13621 (PMC10632077; doi:10.1111/crj.13621)

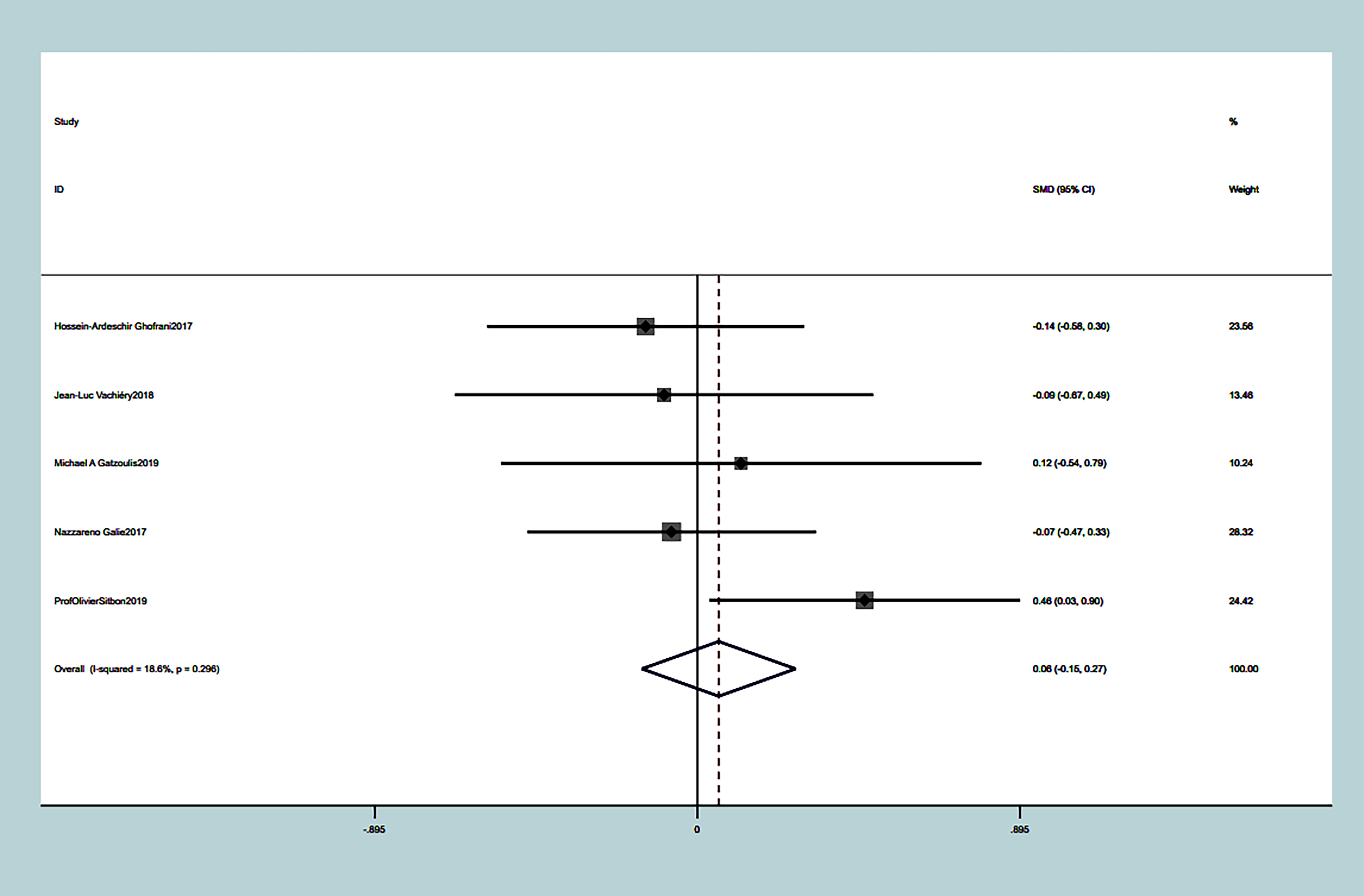

Supplement: Supplementary file 1 — Figure S1. Meta analysis of the effects of macitentan vs placebo on mRAP. [file CRJ-17-1117-s011.tif]

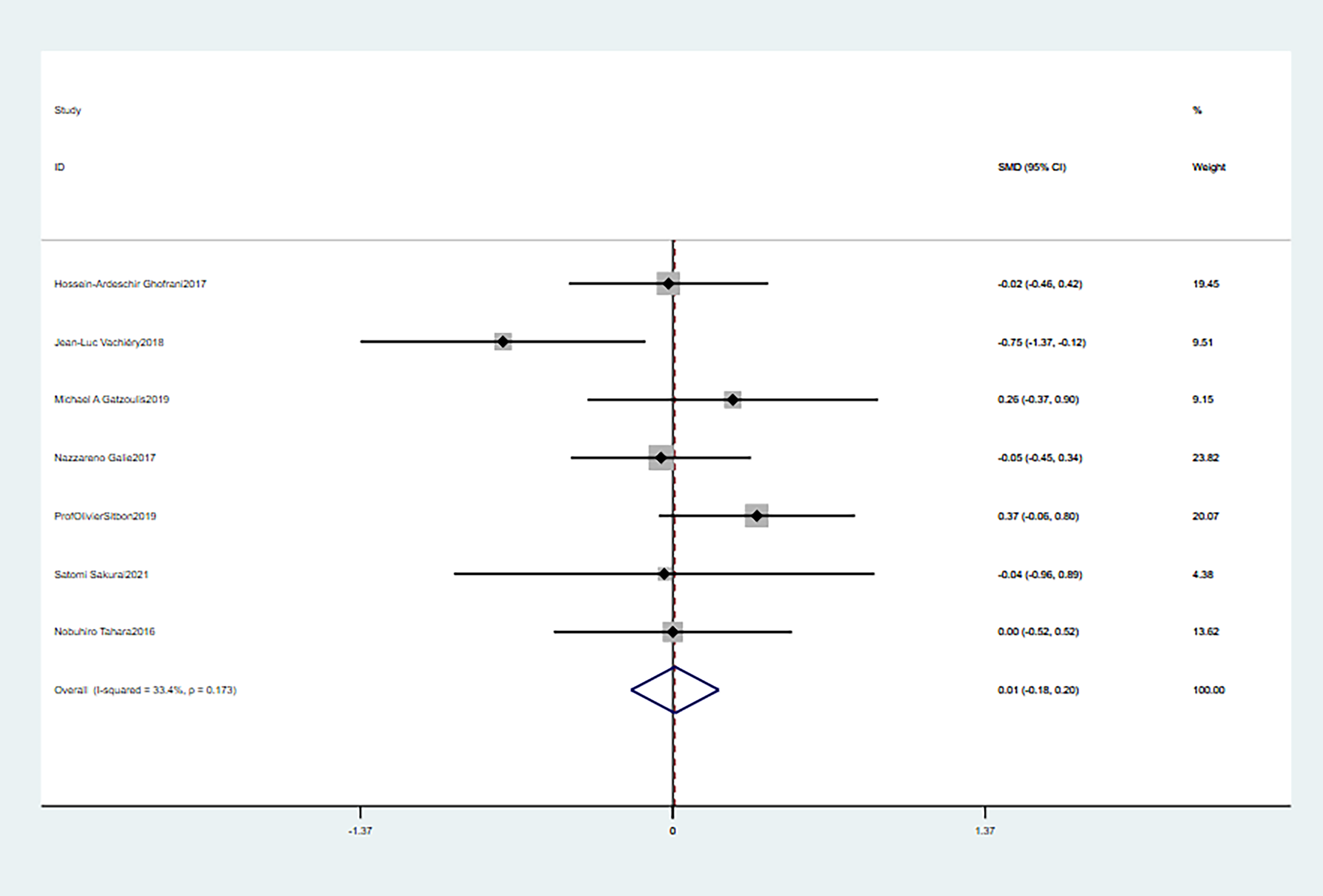

Supplement: Supplementary file 2 — Figure S2. Meta analysis of the effects of Follow‐up vs baseline on mRAP. [file CRJ-17-1117-s008.tif]

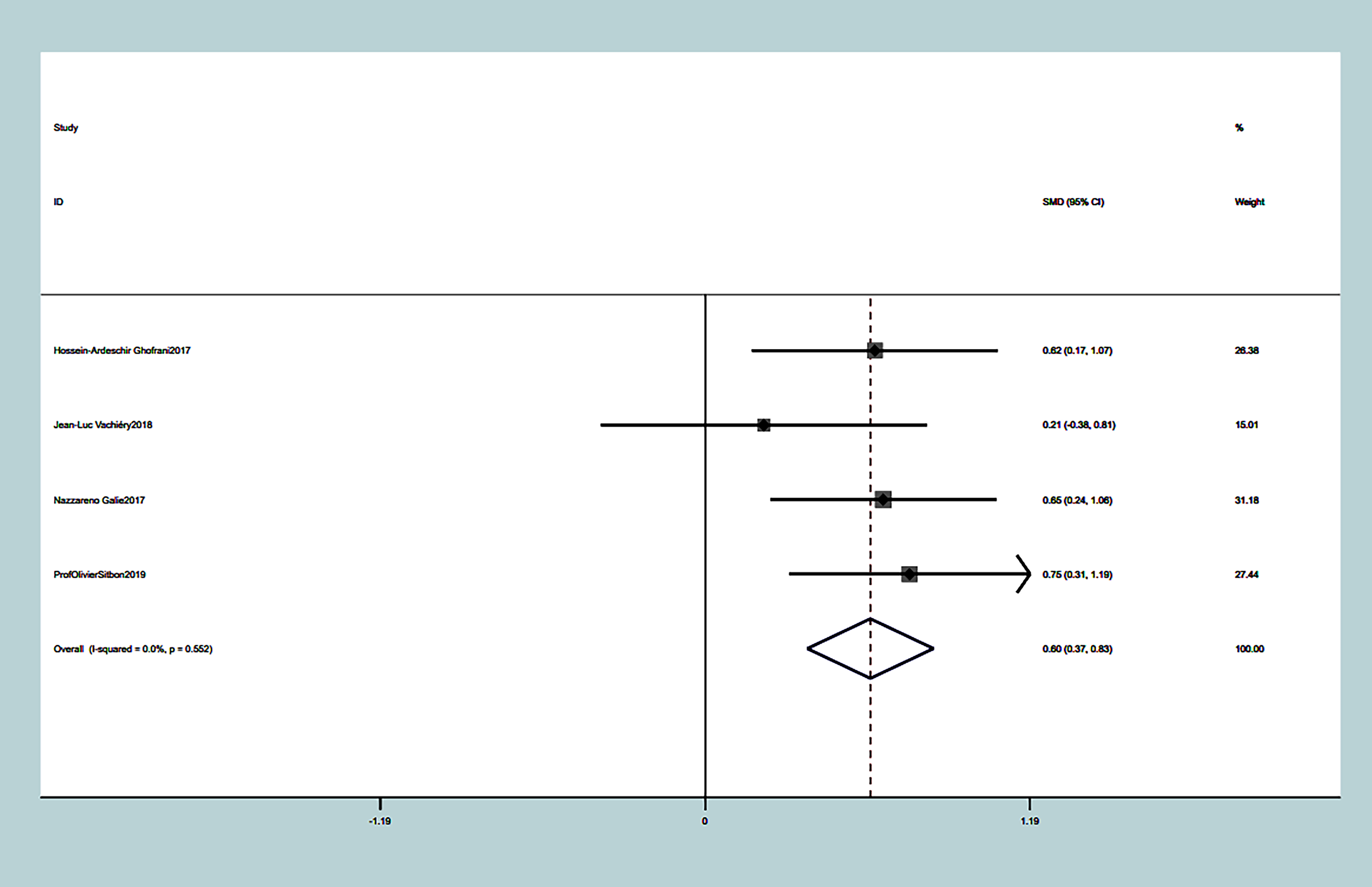

Supplement: Supplementary file 3 — Figure S3. Meta analysis of the effects of macitentan vs placebo on CI. [file CRJ-17-1117-s010.tif]

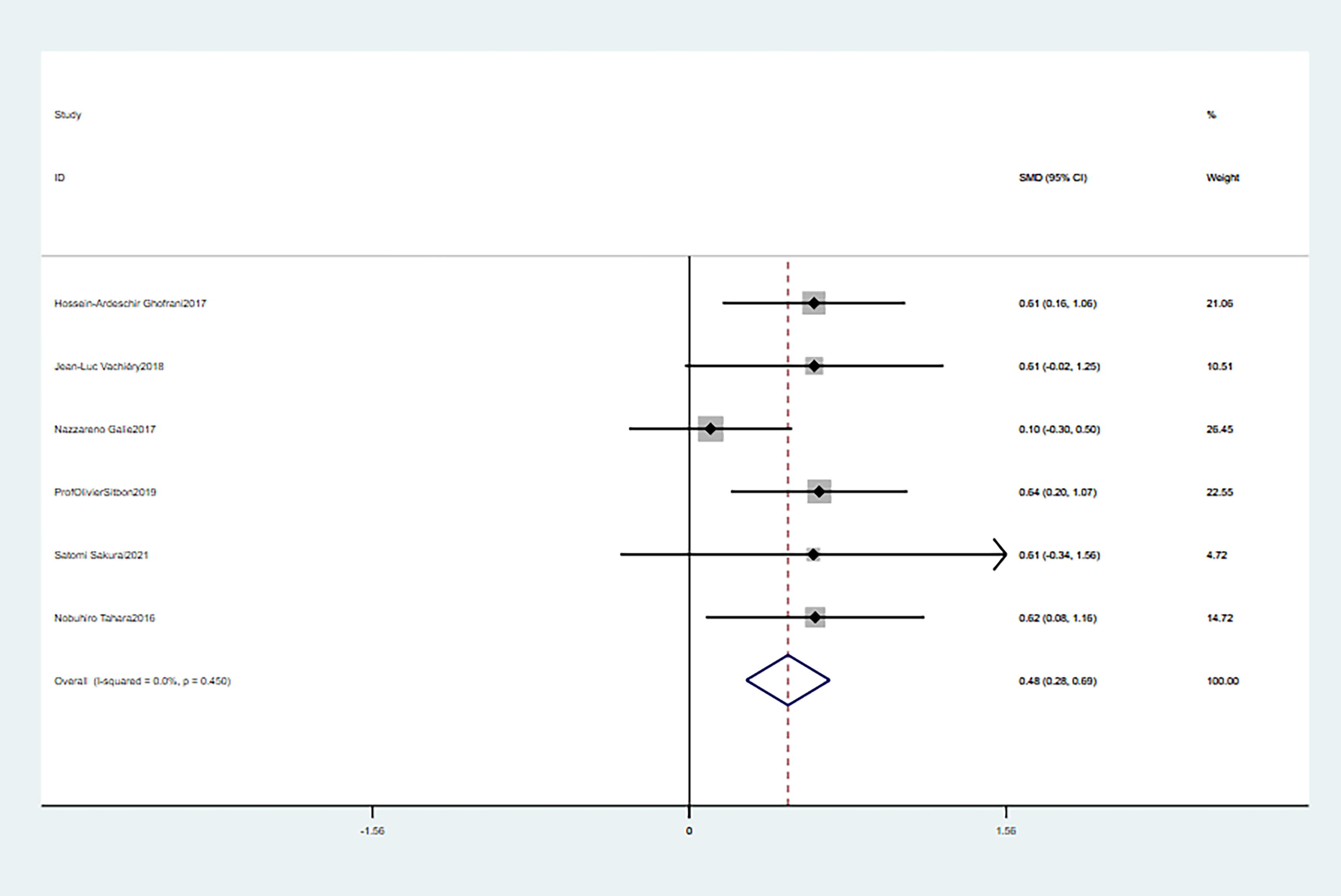

Supplement: Supplementary file 4 — Figure S4. Meta analysis of the effects of Follow‐up vs baseline on CI. [file CRJ-17-1117-s001.tif]

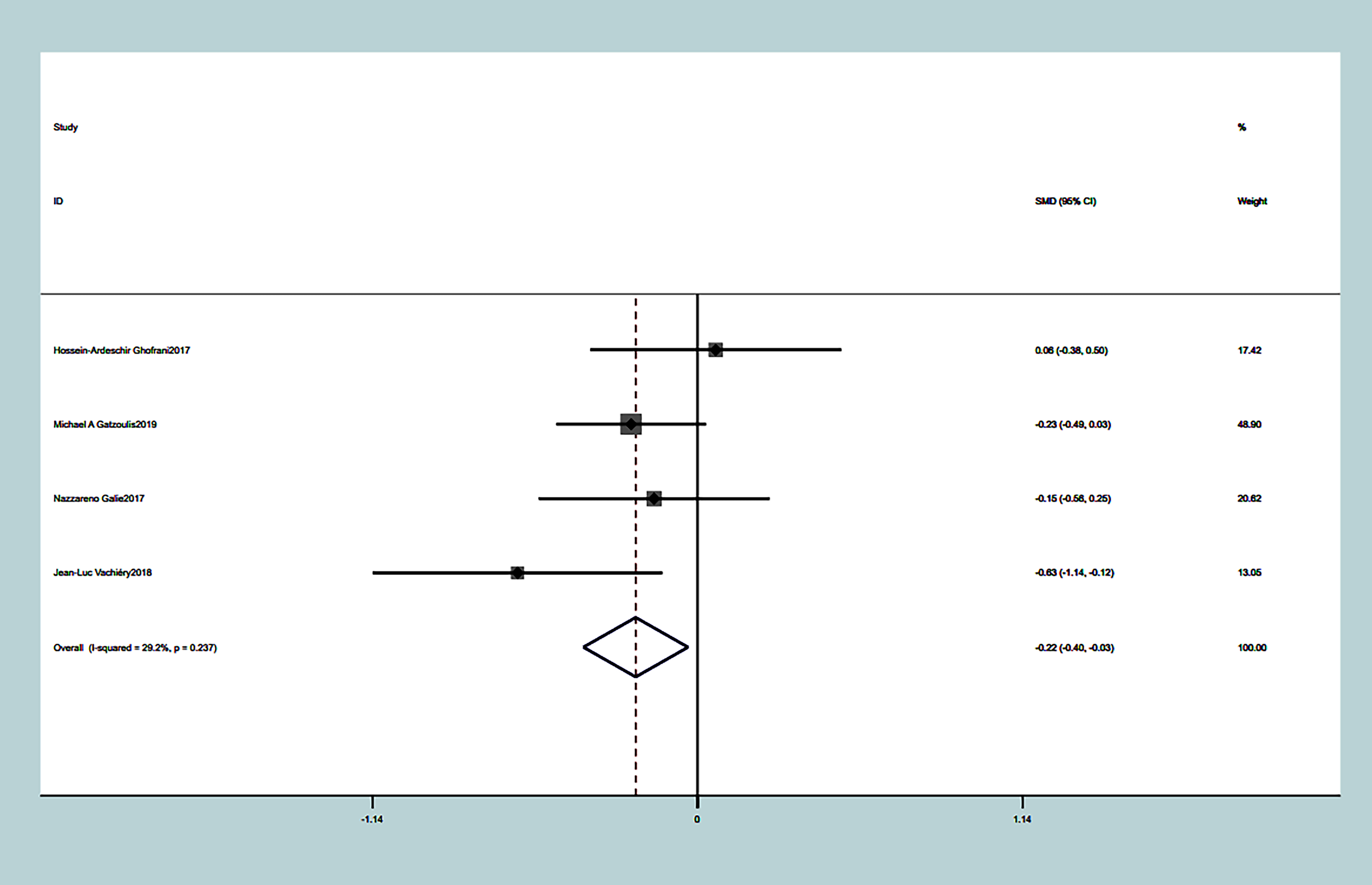

Supplement: Supplementary file 5 — Figure S5. Meta analysis of the effects of macitentan vs placebo on NT‐proBNP. [file CRJ-17-1117-s004.tif]

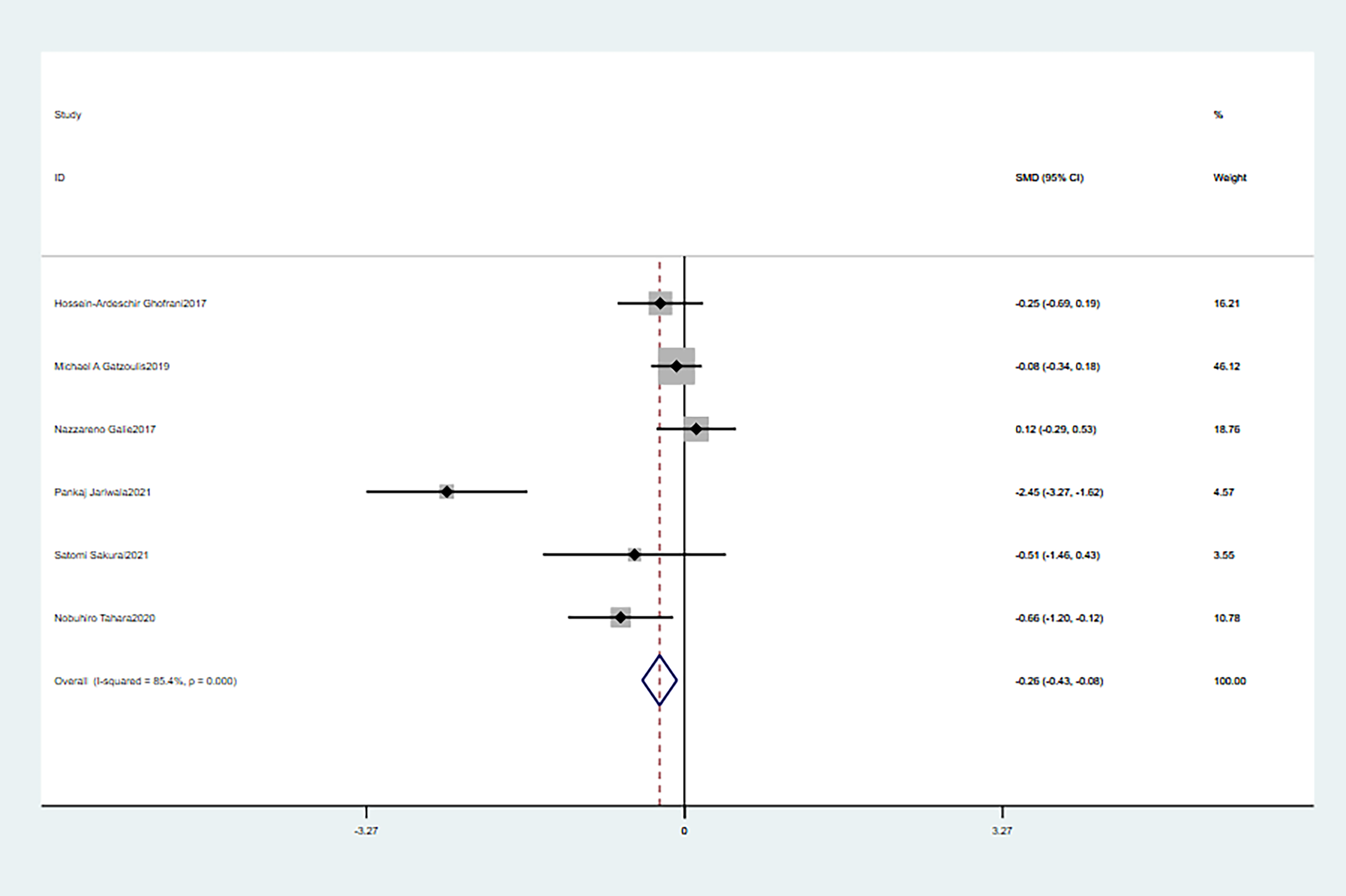

Supplement: Supplementary file 6 — Figure S6. Meta analysis of the effects of Follow‐up vs baseline on NT‐proBNP. [file CRJ-17-1117-s002.tif]

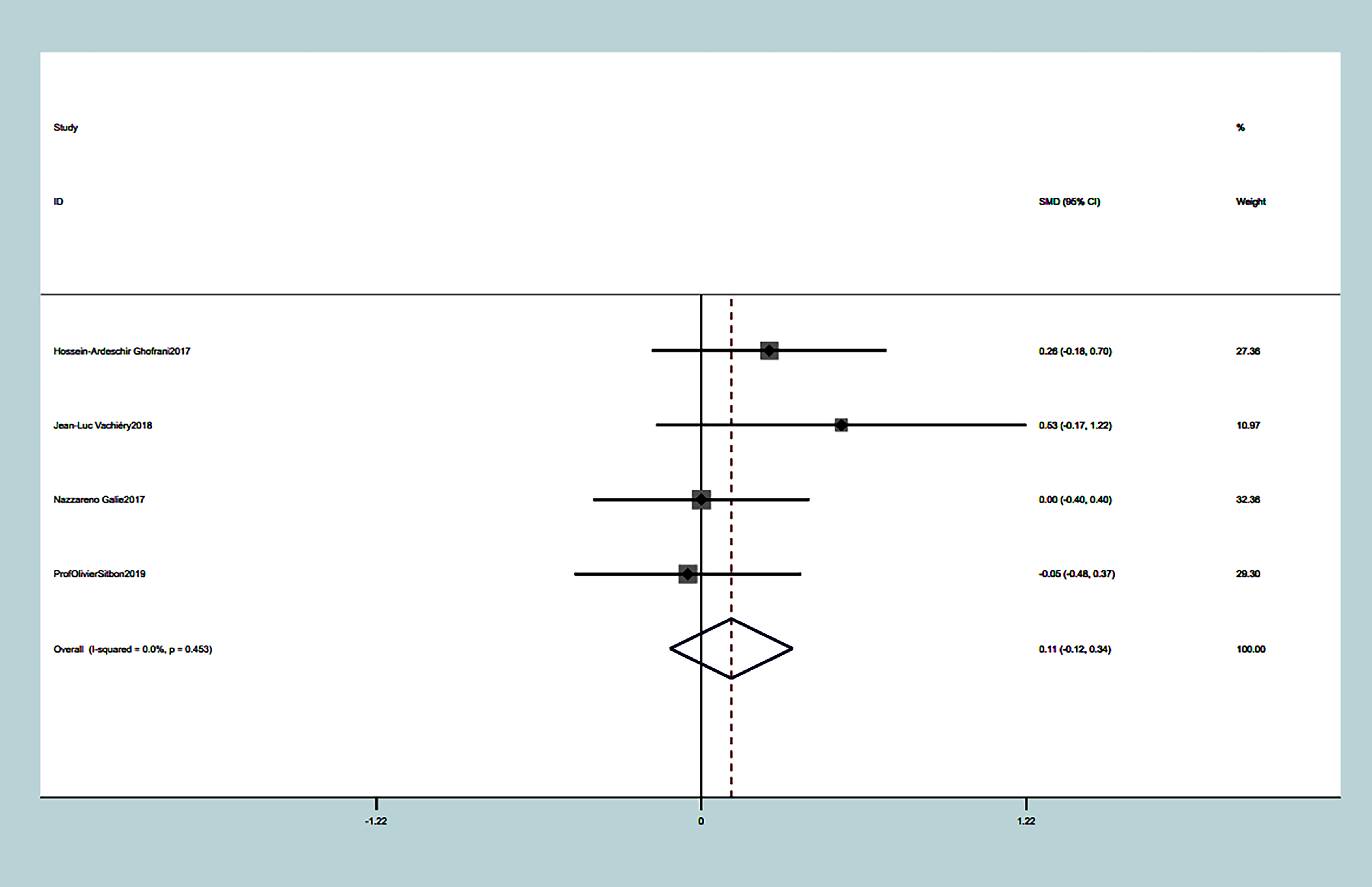

Supplement: Supplementary file 7 — Figure S7. Meta analysis of the effects of macitentan vs placebo on SVO2. [file CRJ-17-1117-s005.tif]

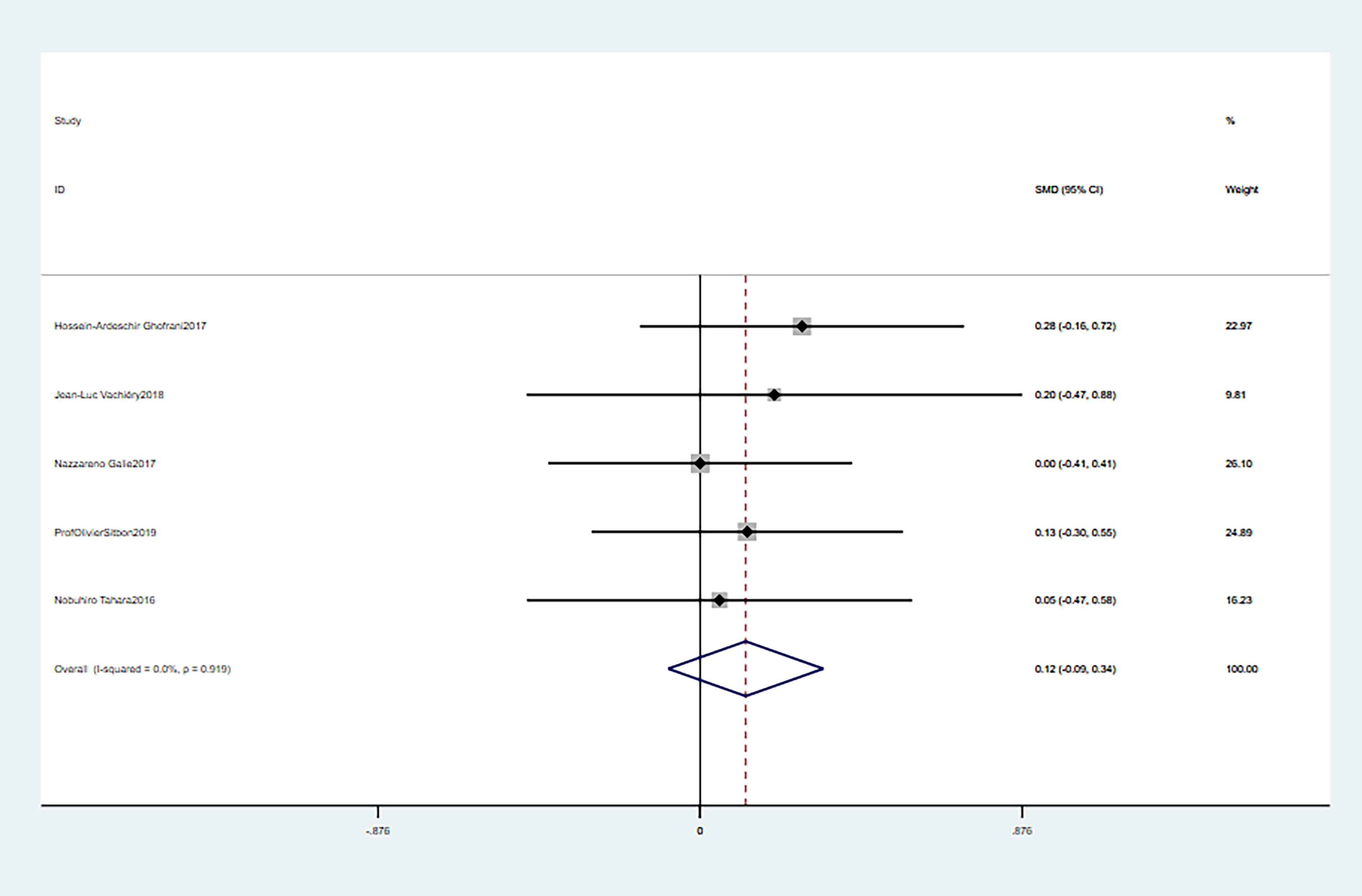

Supplement: Supplementary file 8 — Figure S8. Meta analysis of the effects of Follow‐up vs baseline on SVO2. [file CRJ-17-1117-s006.tif]
