# Supplementary material for: Efficacy and safety of macitentan for pulmonary hypertension: A meta‐analysis
Source: Clin Respir J. 2023 Jul 10;17(11):1117–29. doi: 10.1111/crj.13621 (PMC10632077; doi:10.1111/crj.13621)
Supplement: Supplementary file 14 — Supporting info item [file CRJ-17-1117-s012.docx]

**Efficacy and safety of macitentan for pulmonary hypertension: A meta-analysis**

*Dan Du ^1^, Ya-Dong Yuan ^2*^*

*^1^Department of Respiratory and Critical Care Medicine, The Affiliated Hospital of Guizhou Medical University, 28 Guiyi Street,Beijing Road,Yunyan district,Guiyang,Guizhou,550004, China*

*^2^Department of Respiratory and Critical Care Medicine, The Second Hospital of Hebei Medical University, 215 Heping West Road.Xinhua district,Shijiazhuang, Hebei,050000, China*

**Corresponding author: Ya-Dong Yuan, Department of Respiratory and Critical Care Medicine, The Second Hospital of Hebei Medical University, 215 Heping West Road, Shijiazhuang, Hebei, 050000, China, E-mail: yuanyd1108@163.com, Tel No. +8615803210960*

S1.Meta analysis of the effects of macitentan vs placebo on mRAP

S2.Meta analysis of the effects of Follow-up vs baseline on mRAP

S3.Meta analysis of the effects of macitentan vs placebo on CI

S4.Meta analysis of the effects of Follow-up vs baseline on CI

S5.Meta analysis of the effects of macitentan vs placebo on NT-proBNP

S6.Meta analysis of the effects of Follow-up vs baseline on NT-proBNP

S7.Meta analysis of the effects of macitentan vs placebo on SVO2

S8.Meta analysis of the effects of follow-up vs baseline on SVO2

S9.Meta analysis of mortality in PH

S10.Meta analysis of incidence of peripheral edema

S11.Meta analysis of incidence of headache

S12.Meta analysis of incidence of bronchitis

S13.Meta analysis of incidence of anaemic events
